# Piezoelectric Surgery, Er:YAG Laser Surgery and Nd:YAG Laser Photobiomodulation: A Combined Approach to Treat Medication-Related Osteonecrosis of the Jaws (MRONJ)

**DOI:** 10.3390/dj12080261

**Published:** 2024-08-19

**Authors:** Paolo Vescovi, Pierpaolo De Francesco, Ilaria Giovannacci, Jair Carneiro Leão, Antonio Barone

**Affiliations:** 1 Oral Medicine and Oral Surgery Laser Unit, University Centre of Dentistry, Department of Medicine and Surgery, University of Parma, 43125 Parma, Italy; paolo.vescovi@unipr.it (P.V.); ilaria.giovannacci@gmail.com (I.G.); 2 Unit of Dentistry and Oral Surgery, Department of Surgical, Medical and Molecular Pathology and Critical Care Medicine, University of Pisa, Via Roma 67, 56126 Pisa, Italy; antonio.barone@unipi.it; 3 Departamento de Clínica e Odontologia Preventiva, Universidade Federal de Pernambuco, Recife 50670-901, Brazil; jair.leao@ufpe.br

**Keywords:** piezoelectric surgery, Er:YAG laser surgery, Nd:YAG laser photobiomodulation, MRONJ

## Abstract

Medication-related osteonecrosis of the jaw (MRONJ) is a drug complication that can occur in patients taking antiresorptive or antiangiogenic drugs. Although it is a well-documented disease, there is no widely accepted treatment. However, several therapeutic approaches have been proposed. The surgical approach in many advanced cases appears inevitable; however, the results are not yet defined and predictable. This study aimed to propose a combined surgical approach with a piezoelectric device and laser (Er:YAG for bone ablation and Nd:YAG laser for photobiomodulation) in a young patient with breast cancer and bone metastasis under denosumab treatment, affected by spontaneous stage 3 MRONJ with maxillary sinus involvement. The patient under study reported no post-operative discomfort, with painkiller intake limited to the day after surgery. Total mucosal healing was observed without recurrences for more than 4 years after surgery. According to the results of our preliminary study, a combined surgical approach using a piezoelectric device and laser therapy is effective in managing patients affected by MRONJ, leveraging the clinical and biological advantages of these different techniques.

## 1. Introduction

In 2003 [1], the occurrence of osteonecrosis of the jaw bones (ONJ) was first associated with treatment with a specific drug class, bisphosphonates (BFs). For this reason, the first denomination was Bisphosphonate Osteo-Necrosis (BON). In 2014, there was a change in nomenclature, as it was observed that there are several drug classes that may be associated with the occurrence of this adverse event, such as Densoumab, Sunitibib, Bevacizumab, and biological immunomodulators (e.g., Infliximab, Adalimumab, Rituximab, or mTOR inhibitors). Therefore, the term medication-related osteonecrosis of the jaws (MRONJ) is used [2]. It is now known that the presence of different risk factors is closely related to the evolution of MRONJ, which can be distinguished into the categories “drug-dependent” or “patient-dependent” [3,4] and includes dental procedures such as extractions, implant placement, or local trauma. However, it was observed that the presence of concomitant infections could be the key risk factor for the development of MRONJ. In fact, a study carried out by Nicolatou-Galitis O. et al. [5] suggested that osteonecrosis may already be present at the time of extraction and be associated with local diseases, such as periodontitis or periapical infection; these non-surgery-triggered forms should be considered by oncologists, hematologists, and general physicians, who are advised to inform their patients regarding the importance of preventive dental protocols, in order to control the possible causes of osteonecrosis not related to invasive dental procedures [6,7]. Although it is a well-known, potentially painful and even debilitating condition, proper treatment remains a challenge for dentists, maxillofacial surgeons, and oncologists, as it can drastically affect the quality of life of patients [8]. Several therapeutic approaches have been proposed in the literature over the years [9,10,11,12,13,14,15,16]; however, there are few long-term follow-up retrospective studies available involving multiple patients. In addition, given the severity of the disease, there are no randomized trials with different treatment approaches [17]. However, a surgical approach that aims to selectively remove necrotic bone seems to guarantee better clinical outcomes both in terms of improving symptoms and achieving complete healing [18,19,20], even in early stages of the disease [21]. In this study, we evaluated the effectiveness of a therapeutic treatment combining the benefits of piezoelectric surgery and laser therapy in patients diagnosed with MRONJ according to the AAOMS classification [22]:

- Patients at risk: No apparent necrotic bone in asymptomatic patients treated with IV or oral antiresorptive therapy.
- Stage 0 (non-exposed bone variant): Patients with no clinical evidence of necrotic bone but who present with non-specific symptoms or clinical and radiographic findings.
- Stage 1: Exposed and necrotic bone or fistula that probes the bone in asymptomatic patients with no evidence of infection/inflammation.
- Stage 2: Exposed and necrotic bone, or fistula that probes the bone, with evidence of infection/inflammation. The patients were symptomatic.
- Stage 3: Exposed and necrotic bone or fistulae that probe into the bone, with evidence of infection, and one or more of the following:
  1. Exposed necrotic bone extending beyond the region of alveolar bone
  2. Pathologic fracture
  3. Extraoral fistula
  4. Oral–antral/oral–nasal communication
  5. Osteolysis extending to the inferior border of the mandible or sinus floor

This combined technique makes it possible to exploit the benefits of piezosurgery, such as high cutting precision and reduced trauma on soft tissue, combined with the bio-stimulating effect of laser therapy.

### 1.1. Ultrasonic Bone Surgery

The piezoelectric device was developed by Italian oral surgeon Tommaso Vercelloti in 1988. Its name is derived from the Greek word “piezein”, meaning pressure [23]. Piezosurgery (or ultrasonic bone surgery) has numerous advantages, including that it achieves high cutting precision in bone tissue, does not damage soft and neurovascular tissues, provides more conservative surgery, facilitates better healing, and reduces post-operative complications [24,25,26]. The basic principle of piezosurgery is based on the formation of ultrasonic micro-vibrations created by the piezoelectric effect, wherein certain ceramics and crystals deform upon electric current passing through them, resulting in oscillations in the ultrasonic frequency [27]. In our study, we used a piezosurgery ultrasonic (Mectron^®^, Mectron Medical Technology, Genoa, Italy) device with a maximum power of 120 W and a working frequency that can vary from 24 KHz to 36 KHz. The piezoelectric device is characterized by a power unit connected to an ergonomic piezoelectric hand piece and titanium-nitride-coated tips of various shapes and designs [28]. Saws have been used to remove necrotic bone and bony irregularities and can have different geometries (e.g., vertical and orthogonal) that allow improved access based on the location of the surgical site. In addition to the preceding clinical features, piezosurgery is characterized by its pronounced bactericidal effect during the cavitation process, which provides a significant improvement in terms of bone and mucosal healing, as it results in an alteration of the bacterial biofilm on the surface of the bone and increases the anti-microbial efficacy further than antibiotic release alone [29,30].

### 1.2. Laser Nd:YAG

The effectiveness of different laser wavelengths in the management of MRONJ patients is widely documented [21,31,32]. Nd:YAG laser photobiomodulation, also known as Low-Level Laser Therapy (LLLT), improves and accelerates wound healing [33]. Photobiomodulation results in increased cellular ATP synthesis, reduced oxidative stress, and increased production of growth factors at the molecular level [34]. The application of LLLT has numerous beneficial effects for the patient, including anti-microbial, anti-inflammatory, and analgesic effects [35]. The numerous therapeutic effects of photobiomodulation suggest that the use of this technique as an adjunctive therapy can significantly improve therapeutic outcomes in both medical and surgical management of MRON [36].

### 1.3. Laser Er:YAG

The Er:YAG laser (2940 nm) (Fidelis Plus, Fotona, Ljubljana, Slovenia) (parameters: 300 mJ, 30 Hz, fluence of 60 J/cm^2^) can be used for the treatment of hard and soft tissues of the oral cavity [37]. Each spot of the laser induces vaporization of 0.1 mm of tissue that allows for the gradual and safe ablation of necrotic areas until the bleeding bone is reached. In addition, the formation of microspots enables the promotion of vascularization, healing, and soft tissue adhesion to the bone with bactericidal and bio-stimulatory effects [16,38].

## 2. Materials and Methods

At the Centre of Oral Medicine and Laser Surgery, University of Parma, Italy, a 45-year-old female patient was diagnosed with MRONJ according to the American Association of Oral and Maxillofacial Surgeons diagnostic criteria [2]:

- Current or previous treatment with antiresorptive or antiangiogenic agents.
- Exposed bone or bone that can be probed through an intra-oral or extraoral fistula in the maxillofacial region that has persisted for more than 8 weeks.
- No history of radiation therapy in the jaws or obvious metastatic disease in the jaws.

**Figure 1:**
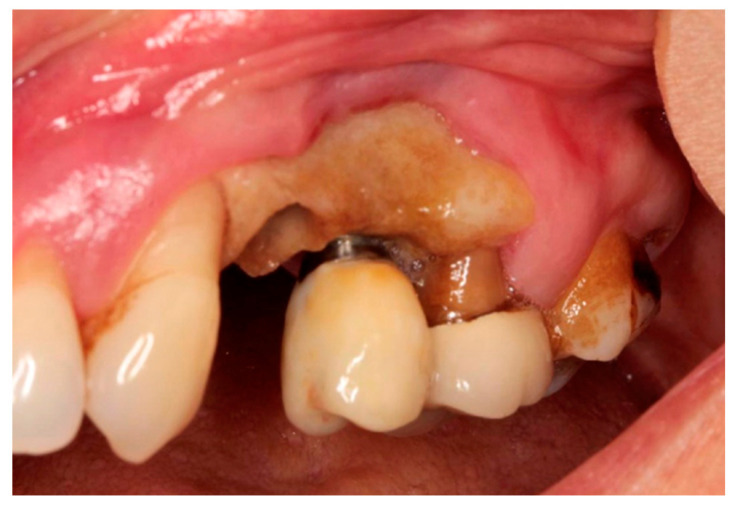
Clinical examination.

**Figure 2:**
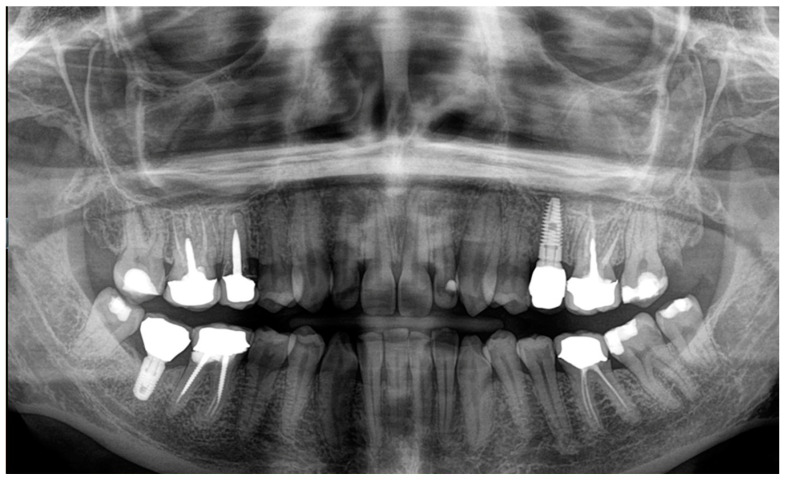
Panoramic radiography.

**Figure 3:**
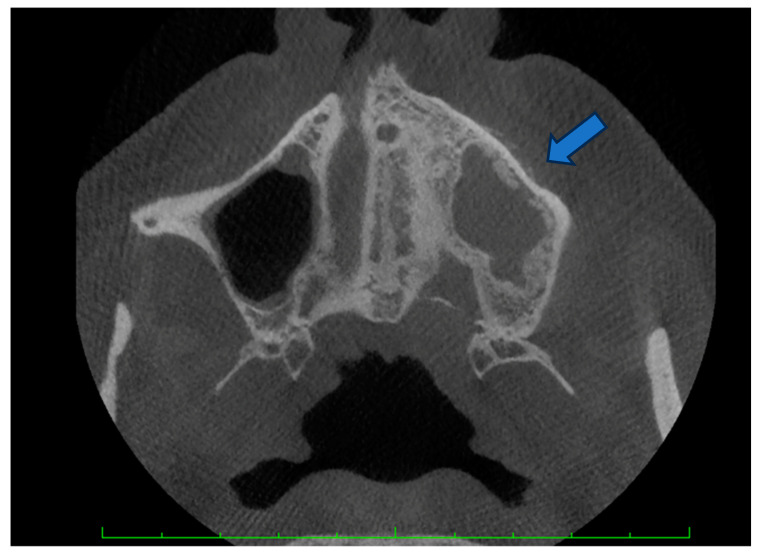
CBCT radiography: maxillary sinus involvement and cortical erosion. The arrow indicates the point at which bone resorption is appreciable.

The patient with breast cancer with bone metastases under denosumab treatment was affected by spontaneous Stage 3 MRONJ with left maxillary sinus involvement, which resulted in the spontaneous loss of tooth element 2.4. Clinical examination showed the presence of a large area of exposed necrotic bone, with signs of infection and the involvement of dental implants in zone 2.5 (Figure 1). Panoramic radiography was prescribed as a first-level examination, followed by a three-dimensional diagnostic technique, such as cone beam computed tomography (CBCT), to more accurately evaluate the actual extension of the osteonecrotic process (Figure 2 and Figure 3).

### Treatment Protocol

**Figure 4:**
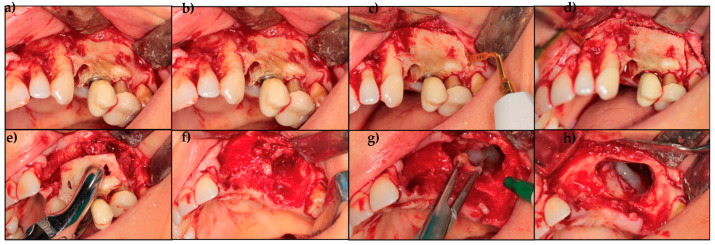
Surgery procedure: (**a**,**b**) mucoperisotal flap elevation; (**c**,**d**) partial osteotomy performed using a piezoelectric device; (**e**) necrotic bone removal; (**f**) surgical site evaluation; (**g**,**h**) infected tissue removal and sinus evaluation.

**Figure 5:**
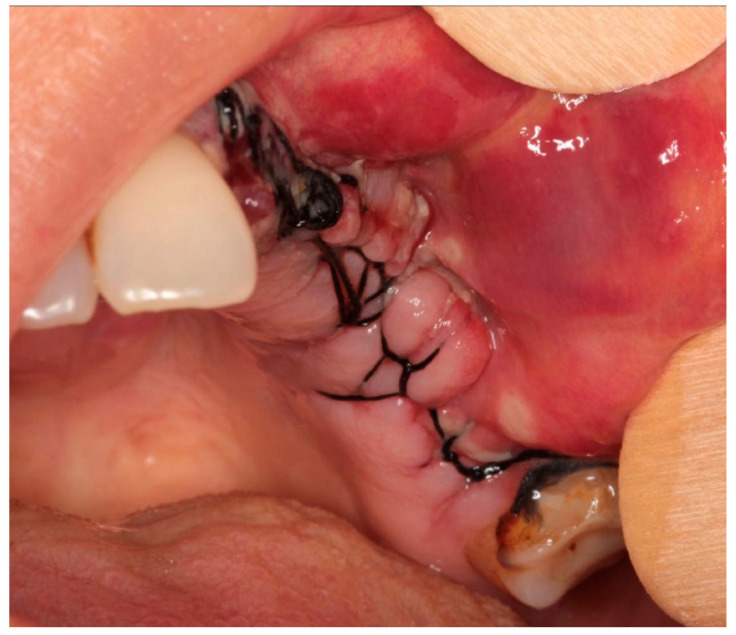
Tension-free wound closure was achieved using continuous sutures.

**Figure 6:**
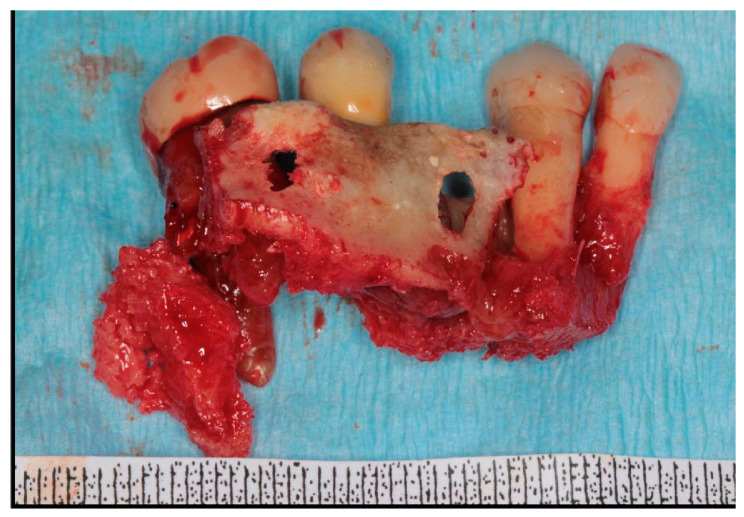
Removed necrotic bone.

After stopping Denosumab 6 months earlier, the patient was initially treated with drug therapy. Medical therapy included oral amoxicillin (1 g × 2/day) and oral metronidazole (250 mg × 2/day) for 3 weeks and mouthwashes with chlorhexidine and hydrogen peroxide two or three times a day. Clinical examination of the MRONJ site was performed 2 weeks after antibiotic discontinuation. After discontinuation of the drug therapy for one month, a second medical therapy was prescribed with the same pharmacological modalities. As complete healing of the osteonecrotic site was not achieved, a decision was made for surgical treatment. Informed consent was obtained for the surgical intervention. Finally, a third cycle of drug therapy was prescribed seven days before surgery and continued for two weeks. The patient was treated surgically under local anesthesia, during which 2.2, 2.3, 2.6 and a dental implant in position 2.5 were extracted. After performing a mucoperiosteal flap, partial osteotomy was performed using a Piezoelectric device. Curettage of the maxillary sinus was also performed, in addition to vaporization of necrotic bone areas until the bleeding bone was reached, and corticotomy of the surrounding clinically healthy bone was performed with an Er:YAG laser (2940 nm) (Fidelis Plus, Fotona, Ljubljana, Slovenia, 300 mJ, 30 Hz, fluence: 60 J/cm^2^). Bone fragments and spikes were eliminated to obtain a smooth surface, avoid local trauma, and facilitate soft tissue healing (Figure 4). The surgical site was irrigated with 10% iodopovidone solution, and tension-free wound closure was achieved using continuous sutures (Figure 5). LLLT applications using a Nd:YAG laser (1064 nm FidelisPlus, Fotona, Ljubljana, Slovenia, 1.25 W, 15 Hz) were performed, starting on the day of surgery and continuing twice a week for four weeks. Each of the LLLT applications was performed with the Nd:YAG laser used at 1.25 W power and 15 Hz frequency and with 320 µm of fibre diameter. Laser light was used in non-focused mode, at 2 mm from the tissue, for 1 min (power density 268.57 W⁄cm^2^), and repeated five times. The removed necrotic bone was analyzed histologically, and the diagnosis of osteonecrosis was confirmed, excluding the presence of metastasis (Figure 6). Use of chlorhexidine 1% gel 4 times daily for two weeks and non-steroidal anti-inflammatory drugs (NSAIDs) (if necessary) were prescribed as post-operative medical therapy. The sutures were removed 3 weeks later. The patient restarted antiresorptive therapy with Denosumab approximately 40 days after surgery.

## 3. Results

**Figure 7:**
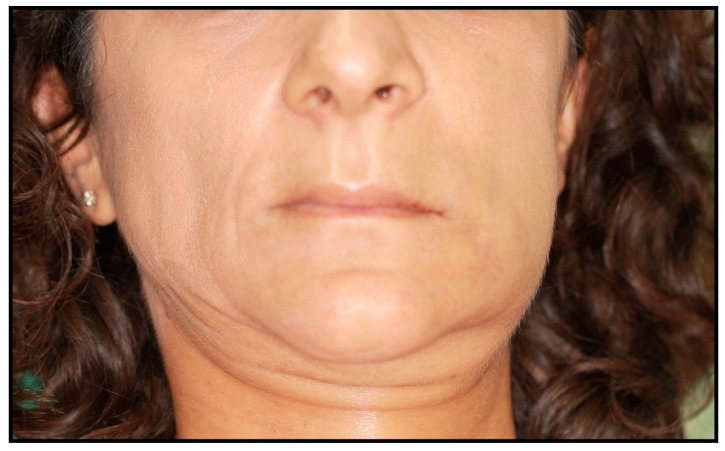
Image at 1-day follow-up.

**Figure 8:**
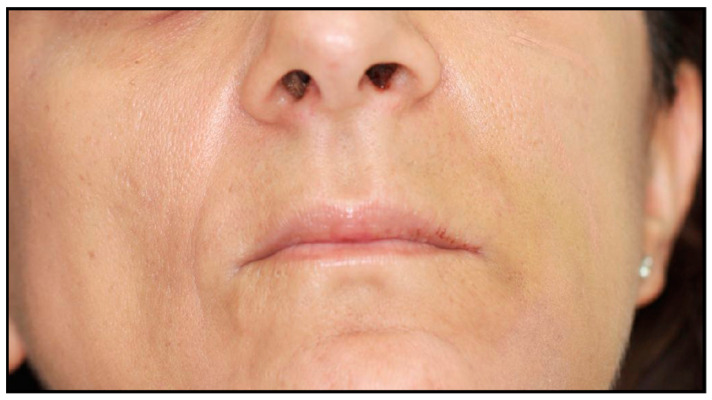
Image at 3-day follow-up.

**Figure 9:**
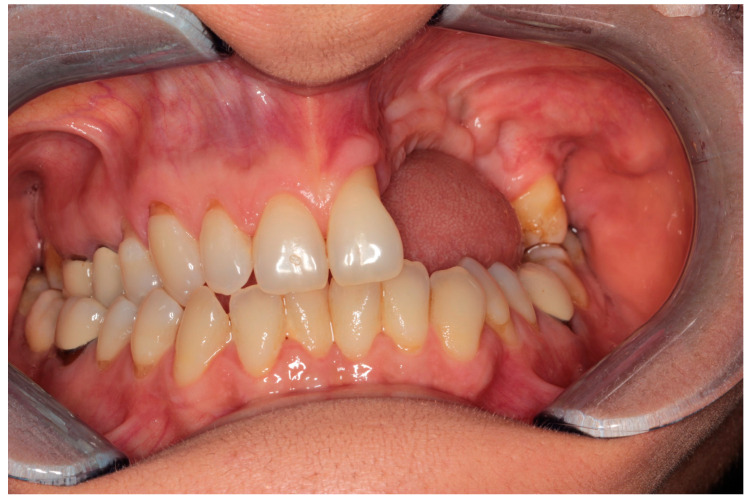
Complete mucosal healing: 3-month follow-up.

**Figure 10:**
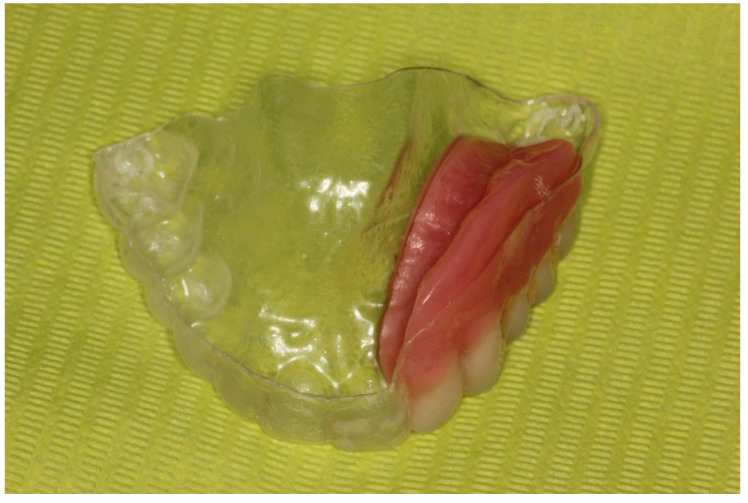
Removable partial prosthesis.

**Figure 11:**
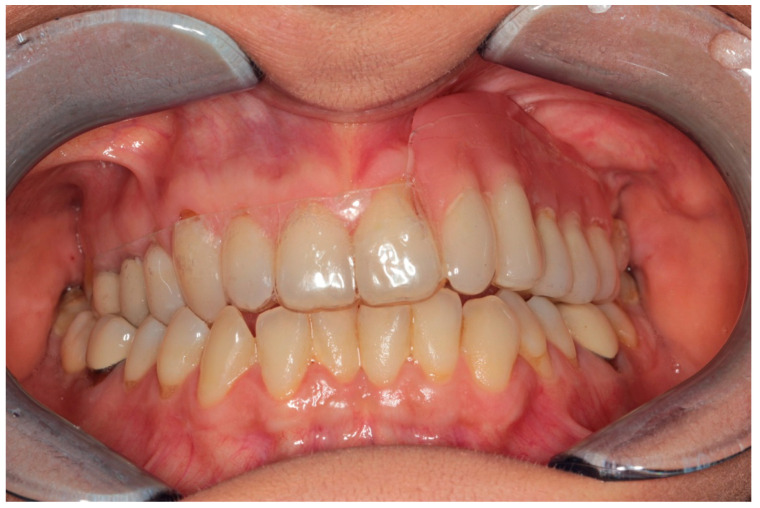
Intra-oral photo.

**Figure 12:**
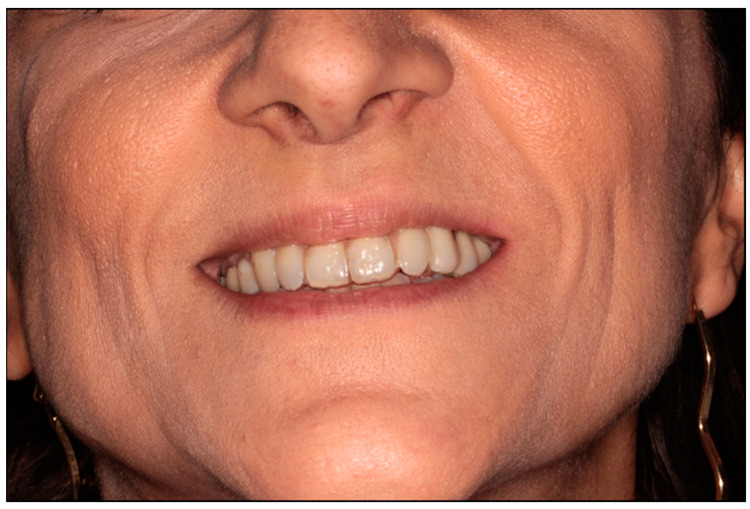
Extra-oral photo.

The patient was treated according to the clinical protocol of Parma’s School for the management of MRONJ, which consists of two cycles of antibiotic therapy and laser photobiomodulation applications first, and a surgical approach second, if complete healing is not achieved. The patient reported no post-operative discomfort, and painkiller intake was limited to the day after surgery (Figure 7 and Figure 8). Complete mucosal healing without signs of infection was achieved one month after surgical intervention. During the post-operative follow-up, the patient visited weekly during the first month, twice a month for the following two months, and once a month for the following six months. After 3 months, there were no signs of disease recurrence, and a removable partial prosthesis was created to restore masticatory and esthetic function (Figure 9, Figure 10, Figure 11 and Figure 12). Total mucosal healing was observed without recurrences for more than 4 years after surgery follow-up. 

## 4. Discussion

MRONJ is a drug complication that was initially exclusively associated with bisphosphonate (BF) treatment. However, an increase in osteonecrosis associated with non-bisphosphonate drugs has been observed. In 2010, the first Denosumab-related osteonecrosis of the jaw (DRONJ) was recorded [39]. Denosumab is an anti-absorbative drug used for the treatment of oncological patients with bone metastases or osteometabolic pathologies (e.g., osteoporosis, Paget’s disease, rheumatoid arthritis, or osteogenesis imperfecta) that guarantee a significant increase in bone mineral density and a reduction in the risk of the occurrence of skeletal-related events (SREs) [40]. It is a human monoclonal IgG2 antibody that selectively binds to the receptor activator of nuclear factor-κB ligand (RANK-L), which prevents RANKL from binding to its receptor RANK (Receptor activator of nuclear factor kappa-Β) on the surface of osteoclasts, blocking their maturation, function and survival [41]. According to the most recent data, Denosumab is associated with a significantly higher risk of developing MRONJ when compared to zoledronate at 1 and 3 years of treatment [42]. The main clinical difference between BRONJ and DRONJ is the latency between the initiation of therapy and the onset of the lesion; DRONJ occurs earlier [43]. Furthermore, the risk would further increase in the event of a therapeutic switch from BPs to Denosumab [44]. Several therapeutic approaches have been proposed for the treatment of MRONJ (Table 1). Although it is a well-known condition, there is a lack of well-designed prospective clinical trials evaluating the proper management of patients suffering from this drug complication, and there is also a discordance in the parameters (e.g., patient QoL scales, mucosal healing, improvement of symptoms, and complete healing) for evaluating the recovery of different studies [45]. The surgical removal of necrotic bone provides better results than the pharmacological approach alone [20]. In addition, the surgical management of earlier stages of the disease allows for the possibility of minimally invasive surgery under local anesthesia and a reduction in post-operative complications [21,46]. The use of laser therapy ensures more successful results than traditional surgery for the management of MRONJ because of the distinct bactericidal and photobiomodulatory effects that allow for the more rapid healing of treated tissues [31].

Currently, there is no clear treatment protocol for the management of MRONJ. The aim of our study was to combine the clinical and biological advantages of piezoelectric surgery and laser therapy to ensure a less invasive surgery and reduce post-operative complications. Piezosurgery is a technique that has been used extensively in oral surgery and the dental field for many years [23]. The piezoelectric surgical device is characterized by physical phenomena in which 26,000 to 38,000 oscillating micro-movements occur with high cutting precision, which allows for very precise osteotomy while avoiding damage to the surrounding soft tissues and maintaining an area of vital and highly reactive bone along the cut [59]. In addition, the cavitation effect ensures higher visibility, removes debris from the cutting area, and provides excellent hemostasis control [23]. However, the distinctive feature for which we decided to use the piezoelectric device for the treatment of MRONJ is its pronounced bactericidal effect, which allows an alteration of the bacterial biofilm present on the bone surface [27]. Studies by Blus et al. [24,60], showed how the cavitation effect produced an antibacterial effect in treating diabetic feet by placing implants in infected sites and decreasing the number of surviving colonies of Escherichia coli and Bacillus subtilis in vitro. Thus, an enhancement in the effectiveness of antibiotic therapy could be due to the alteration of the bacterial biofilm [24]. The most absolute contraindication for piezosurgery is the use of pacemakers [23].

The clinical protocol of Parma’s School for MRONJ management consists firstly of a conservative approach, and secondly, surgical treatment:

1. Antiseptic therapy based on 0.2% clorexidine rinses and hydrogen peroxide 3 times a day in association with antibiotic therapy (amoxicillin 1 g/twice a day + metronidazole 500 g/twice a day) and photobiomodulation laser once a week for five weeks;
2. Suspension for 1 month;
3. Second cycle of antiseptic therapy with antibiotics and photobiomodulation lasers;
4. If complete healing is not achieved, patients are treated with surgical laser assistance.

Initially, conservative treatment allows for clinical improvement, with the possibility of performing less invasive surgery. Furthermore, not all patients can be treated surgically due to the severity of the underlying disease; therefore, the treatment is palliative and not curative, with the aim of improving the quality of life.

The effectiveness of laser therapy in the management of patients with MRONJ has been widely documented [31]. The application of a low-intensity laser (LLLT) using Nd:YAG laser (1064 nm FidelisPlus, Fotona-Slovenia 1.25 W, 15 Hz), to employ a technique also known as photobiomodulation, has been successfully described as an auxiliary treatment in the medical or surgical management of MRONJ [35,61]. From a molecular point of view, we see the absorption, at the mitochondrial level, of photodynamic energy by chromophores (e.g., melanin, cytochrome-c oxidase, and hemoglobin), which are converted into metabolic energy. This results in several beneficial outcomes for the patient, such as analgesic, anti-inflammatory, anti-edema, neoangiogenetic, and anti-microbial effects, and finally, a stimulation of the immune response [31,33,61]. The aim of surgical therapy is the complete removal of the tissue macroscopically involved in the disease, and achievement of healthy tissue that allows stable healing over time [20]. One of the techniques to increase the predictability of surgery is auto-fluorescence, which allows us to distinguish necrotic bone, which appears hypo-fluorescent, from healthy bone, which does not [62]. The Er:YAG laser (2940 nm) can produce a gradual and safe ablation of necrotic areas with anti-microbial properties and bio-stimulant effects [63]. In addition, microspot formation facilitates vascularization and adhesion of soft tissues to the bone, resulting in better post-operative pain management and faster healing [39]. Considering the clinical advantages of the Er:YAG laser, its association with auto-fluorescence is very advantageous in the surgical management of patients with MRONJ [38]. The combined therapeutic approach proposed in this study makes it possible to exploit the advantage of ultrasonic bone surgery, which is that it is possible to perform the first surgical phase, in which large osteotomies can be performed, with a faster cutting speed than the Er:YAG laser, while maintaining the biological and physical advantages over conventional surgical systems (burrs and saws). A second surgical phase allows the use of the Er:YAG laser to vaporize the necrotic bone until the bleeding bone is reached, ensuring a bio-stimulating and minimally invasive effect.

## 5. Conclusions

In conclusion, our innovative approach utilizing a combination of piezoelectric surgery, Er:YAG surgery, and Nd:YAG laser photobiomodulation in the treatment of medication-related osteonecrosis of the jaws achieved an encouraging clinical result. The synergistic effects of these modalities not only addressed the pathological aspects of this disease, but may also minimize post-operative discomfort. Furthermore, the absence of recurrence observed in our clinical case underscores the potential long-term efficacy and sustainability of this treatment strategy. However, it is necessary to emphasize that this single case report needs further confirmation, and that conducting a randomized controlled trial with a larger cohort of patients would allow for further understanding of the long-term impacts.

## Figures and Tables

**Table 1:** Comparison between different outcomes of MRONJ treatments described in the literature.

Authors	N° Patients	Follow-Up (Months)	Clinical Improvement	Complete Healing
J. Freiberger et al. (2007) [47] Hyperbaric oxygen therapy	16		87.5	12.5%
C.I. Ripamonti et al. (2011) [48] Ozone + antibiotic therapy	24		100%	25%
Migliorati CA et al. (2011) [49]				
No surgery (120 sites)	599	24–48		17.6%
Surgery (debridement) (118 sites)		24–48		17.3%
Surgery (flap and/or resection) (316 sites)		24–48		46.3%
A. Agrillo et al. (2012) [50] Ozone + antibiotic therapy + surgery	94	14.3		60%
Martins et al. (2012) [13]				
CHX + antibiotic therapy	3	6		33.3%
Surgery + antibiotic therapy	5	6		60%
Antibiotic therapy + surgery + PRP + LLLT	14	6		85.7%
Rupel K et al. (2014) [51]				
No surgery	77			23.4%
Surgery (debridement)	236			75.4%
Surgery (traditional techniques)	221			90%
Surgery (laser)	56			89.3%
P. Lopez-Jornet et al. (2016) [52] PRP + surgery	123			86%
F. Goker et al. (2020) [53] Ozone + antibiotic therapy + piezosurgery	14		90%	64.2%
Morishita et al. (2020) [54] Teriparatide therapy	29		75.9%	65.5%
Yüce et al. (2021) [55]				
Surgery + concentrated growth factor (CGF)	14			78.6%
Surgery without CGF	14			57.1%
Sahin et al. (2021) [56] Piezosurgery + leukocyte and platelet-rich fibrin concentrate (L-PRF) +Nd:YAG laser	21			100%
Otsuru et al. (2022) [57] Marginal mandibulectomy Segmental mandibulectomy				
	155			67.1%
	13			92.3%
Parise et al. (2022) [58]				
Surgery + fibrin-rich platelets and leukocytes (L-PRF) + antibiotic therapy	5			80%
Surgery + without L-PRF + antibiotic therapy	7			57%
P. Vescovi et al. (2012) [8]				
G1 (28 sites): medical therapy; G2 (32 sites): medical therapy and LLLT	151		G1: 25% G2: 71.8% G3: 64.7% G4: 81.8% G5: 96.5%	G1: 17.8% G2: 28.1% G3: 64.7% G4: 72.7% G5: 89.6%
G3 (17 sites): medical and conventional surgical therapy				
G4 (33 sites): medical therapy, conventional surgical therapy, and LLLT				
G5 (29 sites): medical therapy, Er:YAG laser surgical therapy, and LLLT				

## Data Availability

Data are available on request from the authors.

## References

[1] Marx R.E. (2003). Pamidronate (Aredia) and zoledronate (Zometa) induced avascular necrosis of the jaws: A growing epidemic. J. Oral Maxillofac. Surg..

[2] Ruggiero S.L., Dodson T.B. (2014). American association of oral and maxillofacial surgeons position paper on medication-related osteonecrosis of the jaw—2014 update. J. Oral Maxillofac. Surg..

[3] AlRowis R., Aldawood A., AlOtaibi M., Alnasser E., AlSaif I., Aljaber A., Natto Z. (2022). Medication-Related Osteonecrosis of the Jaw (MRONJ): A Review of Pathophysiology, Risk Factors, Preventive Measures and Treatment Strategies. Saudi Dent. J..

[4] Izzetti R., Gennai S. (2023). Panoramic Radiography Features of Medication-Related Osteonecrosis of the Jaws (MRONJ). Int. J. Dent..

[5] Nicolatou-Galitis O., Razis E., Galiti D., Galitis E., Labropoulos S., Tsimpidakis A., Sgouros J., Karampeazis A., Migliorati C. (2015). Periodontal disease preceding osteonecrosis of the jaw (ONJ) in cancer patients receiving antiresorptives alone or combined with targeted therapies: Report of 5 cases and literature review. Oral Surg. Oral Med. Oral Pathol. Oral Radiol..

[6] Vescovi P., Campisi G., Fusco V., Mergoni G., Manfredi M., Merigo E., Solazzo L., Gabriele M., Gaeta G.M., Favia G. (2011). Surgery-triggered and non surgery-triggered Bisphosphonate-related Osteonecrosis of the Jaws (BRONJ): A retrospective analysis of 567 cases in an Italian multicenter study. Oral Oncol..

[7] Merigo E., Manfredi M., Meleti M., Corradi D., Vescovi P. (2005). Jaw bone necrosis without previous dental extractions associated with the use of bisphosphonates (pamidronate and zoledronate): A four-case report. J. Oral Pathol. Med..

[8] Vescovi P., Merigo E., Meleti M., Manfredi M., Guidotti R., Nammour S. (2012). Bisphosphonates-related osteonecrosis of the jaws: A concise review of the literature and a report of a single-centre experience with 151 patients. J. Oral Pathol. Med..

[9] Ceponis P., Keilman C., Guerry C., Freiberger J. (2017). Hyperbaric oxygen therapy and osteonecrosis. Oral Dis..

[10] Cavalcante R.C., Tomasetti G. (2020). Pentoxifylline and tocopherol protocol to treat medication-related osteonecrosis of the jaw: A systematic literature review. J. Cranio-Maxillofac. Surg..

[11] Beth-Tasdogan N.H., Mayer B., Hussein H., Zolk O. (2017). Interventions for managing medication-related osteonecrosis of the jaw. Cochrane Database Syst. Rev..

[12] Nisi M., Karapetsa D., Gennai S., Ramaglia L., Graziani F., Gabriele M. (2018). Conservative surgical treatment of medication related osteonecrosis of the jaw (MRONJ) lesions in patients affected by osteoporosis exposed to oral bisphosphonates: 24 months follow-up. J. Cranio-Maxillofac. Surg..

[13] Martins M.A.T., Lascala C.A., Curi M.M., Migliorati C.A., Tenis C.A., Marques M.M. (2012). Association of laser phototherapy with PRP improves healing of bisphosphonate-related osteonecrosis of the jaws in cancer patients: A preliminary study. Oral Oncol..

[14] Suh Y., Patel S., Kaitlyn R., Gandhi J., Joshi G., Smith N.L., Khan S.A. (2019). Clinical utility of ozone therapy in dental and oral medicine. Med. Gas Res..

[15] Stübinger S., Dissmann J., Pinho N.C., Saldamli B., Seitz O., Sader R. (2009). A preliminary report about treatment of bisphosphonate related osteonecrosis of the jaw with Er:YAG laser ablation. Lasers Surg. Med..

[16] Sacco R., Akintola O., Sacco N., Acocella A., Calasans-Maia M.D., Maranzano M., Olate S. (2023). The Use of Human Amniotic Membrane (hAM) as a Treatment Strategy of Medication-Related Osteonecrosis of the Jaw (MRONJ): A Systematic Review and Meta-Analysis of the Literature. Medicina.

[17] Goker F., Grecchi E., Grecchi F., Francetti L., Del Fabbro M. (2021). Treatment of medication-related osteonecrosis of the jaw (MRONJ). A systematic review. Eur. Rev. Med. Pharmacol. Sci..

[18] Campisi G., Mauceri R., Bertoldo F., Bettini G., Biasotto M., Colella G., Consolo U., Di Fede O., Favia G., Fusco V. (2020). Medication-related osteonecrosis of jaws (MRONJ) prevention and diagnosis: Italian consensus update 2020. Int. J. Environ. Res. Public Health.

[19] Graziani F., Vescovi P., Campisi G., Favia G., Gabriele M., Gaeta G.M., Gennai S., Goia F., Miccoli M., Peluso F. (2012). Resective surgical approach shows a high performance in the management of advanced cases of bisphosphonate-related osteonecrosis of the jaws: A retrospective survey of 347 cases. J. Oral Maxillofac. Surg..

[20] El-Rabbany M., Lam D.K., Shah P.S., Azarpazhooh A. (2019). Surgical Management of Medication-Related Osteonecrosis of the Jaw Is Associated With Improved Disease Resolution: A Retrospective Cohort Study. J. Oral Maxillofac. Surg..

[21] Vescovi P., Manfredi M., Merigo E., Guidotti R., Meleti M., Pedrazzi G., Fornaini C., Bonanini M., Ferri T., Nammour S. (2012). Early Surgical Laser-Assisted Management of Bisphosphonate-Related Osteonecrosis of the Jaws (BRONJ): A Retrospective Analysis of 101 Treated Sites with Long-Term Follow-Up. Photomed. Laser Surg..

[22] Ruggiero S.L., Dodson T.B., Aghaloo T., Carlson E.R., Ward B.B., Kademani D. (2022). American Association of Oral and Maxillofacial Surgeons’ Position Paper on Medication-Related Osteonecrosis of the Jaws—2022 Update. J. Oral Maxillofac. Surg..

[23] Kirpalani T., Dym H. (2020). Role of Piezo Surgery and Lasers in the Oral Surgery Office. Dent. Clin. N. Am..

[24] Blus C., Giannelli G., Szmukler-Moncler S., Orru G. (2017). Treatment of medication-related osteonecrosis of the jaws (MRONJ) with ultrasonic piezoelectric bone surgery. A case series of 20 treated sites. Oral Maxillofac. Surg..

[25] Pavlíková G., Foltán R., Horká M., Hanzelka T., Borunská H., Šedý J. (2011). Piezosurgery in oral and maxillofacial surgery. Int. J. Oral Maxillofac. Surg..

[26] Covani U., Barone A. (2007). Piezosurgical Treatment of Unicystic Ameloblastoma. J. Periodontol..

[27] Ealla K.K.R., Thomas M., Akula U., Gajjada N. (2017). Piezosurgery: A boon for modern periodontics. J. Int. Soc. Prev. Community Dent..

[28] Crovace A.M., Luzzi S., Lacitignola L., Fatone G., Lucifero A.G., Vercellotti T., Crovace A. (2020). Minimal invasive piezoelectric osteotomy in neurosurgery: Technic, applications, and clinical outcomes of a retrospective case series. Vet. Sci..

[29] Blus C., Szmukler-Moncler S., Giannelli G., Denotti G., Orrù G. (2013). Use of Ultrasonic Bone Surgery (Piezosurgery) to Surgically Treat Bisphosphonate-Related Osteonecrosis of the Jaws (BRONJ). A Case Series Report with at Least 1 Year of Follow-Up. Open Dent. J..

[30] Kopel M., Degtyar E., Banin E. (2011). Surface acoustic waves increase the susceptibility of Pseudomonas aeruginosa biofilms to antibiotic treatment. Biofouling.

[31] Momesso G.A.C., Lemos C.A.A., Santiago-Júnior J.F., Faverani L.P., Pellizzer E.P. (2020). Laser surgery in management of medication-related osteonecrosis of the jaws: A meta-analysis. Oral Maxillofac. Surg..

[32] Tolentino E.d.S., de Castro T.F., Michellon F.C., Passoni A.C.C., Ortega L.J.A., Iwaki L.C.V., da Silva M.C. (2019). Adjuvant therapies in the management of medication-related osteonecrosis of the jaws: Systematic review. Head Neck.

[33] Choung H.-W., Lee S.-H., Ham A.R., Lee N.R., Kim B., Pang K.-M., Jahng J.W., Lee J.-H. (2019). Effectiveness of low-level laser therapy with a 915 nm wavelength diode laser on the healing of intraoral mucosal wound: An animal study and a double-blind randomized clinical trial. Medicina.

[34] Glass G.E. (2021). Photobiomodulation: The clinical applications of low-level light therapy. Aesthetic Surg. J..

[35] Kalhori K.A., Vahdatinia F., Jamalpour M.R., Vescovi P., Fornaini C., Merigo E., Fekrazad R. (2019). Photobiomodulation in Oral Medicine. Photobiomodulation Photomed. Laser Surg..

[36] Razavi P., Jafari A., Vescovi P., Fekrazad R. (2022). Efficacy of Adjunctive Photobiomodulation in the Management of Medication-Related Osteonecrosis of the Jaw: A Systematic Review. Photobio. Photomed. Laser Surg..

[37] Sasaki K.M., Aoki A., Ichinose S., Yoshino T., Yamada S., Ishikawa I. (2002). Scanning Electron Microscopy and Fourier Transformed Infrared Spectroscopy Analysis of Bone Removal Using Er:YAG and CO_2_ Lasers. J. Periodontol..

[38] Vescovi P., Giovannacci I., Otto S., Manfredi M., Merigo E., Fornaini C., Nammour S., Meleti M. (2015). Medication-Related Osteonecrosis of the Jaw: An Autofluorescence-Guided Surgical Approach Performed with Er:YAG Laser. Photomed. Laser Surg..

[39] Taylor K., Middlefell L., Mizen K. (2010). Osteonecrosis of the jaws induced by anti-RANK ligand therapy. Br. J. Oral Maxillofac. Surg..

[40] Kearns A.E., Khosla S., Kostenuik P.J. (2008). Receptor activator of nuclear factor κB ligand and osteoprotegerin regulation of bone remodeling in health and disease. Endocr. Rev..

[41] Limones A., Sáez-Alcaide L., Díaz-Parreño S., Helm A., Bornstein M., Molinero-Mourelle P. (2011). Denosumab-related osteonecrosis of the jaws. Osteoporos. Int..

[42] Limones A., Sáez-Alcaide L.M. (2020). Medication-related osteonecrosis of the jaws (MRONJ) in cancer patients treated with denosumab VS. zoledronic acid: A systematic review and meta-analysis. Med. Oral Patol. Oral Y Cir. Bucal.

[43] A AlDhalaan N., BaQais A., Al-Omar A. (2020). Medication-related Osteonecrosis of the Jaw: A Review. Cureus.

[44] Otto S., Pautke C., Van den Wyngaert T., Niepel D., Schiødt M. (2018). Medication-related osteonecrosis of the jaw: Prevention, diagnosis and management in patients with cancer and bone metastases. Cancer Treat. Rev..

[45] Vescovi P., Manfredi M., Merigo E., Meleti M. (2008). Early Surgical Approach Preferable to Medical Therapy for Bisphosphonate-Related Osteonecrosis of the Jaws. J. Oral Maxillofac. Surg..

[46] Weber J.B.B., Camilotti R.S., Ponte M.E. (2016). Efficacy of laser therapy in the management of bisphosphonate-related osteonecrosis of the jaw (BRONJ): A systematic review. Lasers Med Sci..

[47] Freiberger J.J., Padilla-Burgos R., Chhoeu A.H., Kraft K.H., Boneta O., Moon R., Piantadosi C. (2007). Hyperbaric Oxygen Treatment and Bisphosphonate-Induced Osteonecrosis of the Jaw: A Case Series. J. Oral Maxillofac. Surg..

[48] Ripamonti C.I., Cislaghi E., Mariani L., Maniezzo M. (2011). Efficacy and safety of medical ozone (O3) delivered in oil suspension applications for the treatment of osteonecrosis of the jaw in patients with bone metastases treated with bisphosphonates: Preliminary results of a phase I-II study. Oral Oncol..

[49] Migliorati C.A., Epstein J.B., Abt E., Berenson J.R. (2011). Osteonecrosis of the jaw and bisphosphonates in cancer: A narrative review. Nat. Rev. Endocrinol..

[50] Agrillo A., Filiaci F., Ramieri V., Riccardi E., Quarato D., Rinna C., Gennaro P., Cascino F., Mitro V., Ungari C. (2012). Bisphosphonate-related osteonecrosis of the jaw (BRONJ): 5 year experience in the treatment of 131 cases with ozone therapy. Eur. Rev. Med. Pharmacol. Sci..

[51] Rupel K., Ottaviani G., Gobbo M., Contardo L., Tirelli G., Vescovi P., Di Lenarda R., Biasotto M. (2014). A systematic review of therapeutical approaches in bisphosphonates-related osteonecrosis of the jaw (BRONJ). Oral Oncol..

[52] Lopez-Jornet P., Perez A.S., Mendes R.A., Tobias A. (2016). Medication-related osteonecrosis of the jaw: Is autologous platelet concentrate application effective for prevention and treatment? A systematic review. J. Cranio-Maxillofac. Surg..

[53] Goker F., Donati G., Grecchi F., Sparaco A., Ghezzo M., Rania V., Rossi C.A., Del Fabbro M. (2020). Treatment of BRONJ with ozone/oxygen therapy and debridement with piezoelectric surgery. Eur. Rev. Med. Pharmacol. Sci..

[54] Morishita K., Yamada S.-I., Kawakita A., Hashidume M., Tachibana A., Takeuchi N., Ohbayashi Y., Kanno T., Yoshiga D., Narai T. (2020). Treatment outcomes of adjunctive teriparatide therapy for medication-related osteonecrosis of the jaw (MRONJ): A multicenter retrospective analysis in Japan. J. Orthop. Sci..

[55] Yüce M.O., Adalı E., Işık G. (2021). The effect of concentrated growth factor (CGF) in the surgical treatment of medication-related osteonecrosis of the jaw (MRONJ) in osteoporosis patients: A randomized controlled study. Clin. Oral Investig..

[56] Şahin O., Akan E., Tatar B., Ekmekcioğlu C., Ünal N., Odabaşı O. (2022). Combined approach to treatment of advanced stages of medication-related osteonecrosis of the jaw patients. Braz. J. Otorhinolaryngol..

[57] Otsuru M., Soutome S., Hayashida S., Rokutanda S., Yanamoto S., Umeda M. (2022). A preliminary clinical study of segmental mandibulectomy on medication-related osteonecrosis of the jaw. J. Dent. Sci..

[58] Parise G.K., Costa B.N., Nogueira M.L., Sassi L.M., Schussel J.L. (2023). Efficacy of fibrin-rich platelets and leukocytes (L-PRF) in tissue repair in surgical oral procedures in patients using zoledronic acid—case–control study. Oral Maxillofac. Surg..

[59] Costa D.L., de Azevedo E.T., Przysiezny P.E., Kluppel L.E. (2021). Use of Lasers and Piezoelectric in Intraoral Surgery. Oral Maxillofac. Surg. Clin. N. Am..

[60] Blus C., Szmukler-Moncler S., Khoury P., Orrù G. (2015). Immediate implants placed in infected and noninfected sites after atraumatic tooth extraction and placement with ultrasonic bone surgery. Clin. Implant. Dent. Relat. Res..

[61] Ghidini G., Manfredi M., Giovannacci I., Mergoni G., Sarraj A., Mureddu M., Giunta G., Bonanini M., Meleti M., Vescovi P. (2017). Medication-related osteonecrosis of the jaw: Risk factors in patients under biphosphonate versus patients under antiresorptive-antiangiogenic drugs. Minerva Stomatol..

[62] Dipalma G., Inchingolo A.D., Piras F., Palmieri G., Pede C.D., Ciocia A.M., Siciliani R.A., Olio F.D., Inchingolo A.M., Palermo A. (2023). Efficacy of guided autofluorescence laser therapy in MRONJ: A systematic review. Eur. Rev. Med. Pharmacol. Sci..

[63] Porcaro G., Amosso E., Scarpella R., Carini F. (2015). Doxycycline fluorescence-guided Er:YAG laser ablation combined with Nd:YAG/diode laser biostimulation for treating bisphosphonate-related osteonecrosis of the jaw. Oral Surg. Oral Med. Oral Pathol. Oral Surg. Oral Med. Oral Pathol. Oral Radiol..

